# Metabolipidomic Analysis in Patients with Obstructive Sleep Apnea Discloses a Circulating Metabotype of Non-Dipping Blood Pressure

**DOI:** 10.3390/antiox12122047

**Published:** 2023-11-27

**Authors:** Lucía Pinilla, Iván D. Benítez, Esther Gracia-Lavedan, Gerard Torres, Olga Mínguez, Rafaela Vaca, Mariona Jové, Joaquim Sol, Reinald Pamplona, Ferran Barbé, Manuel Sánchez-de-la-Torre

**Affiliations:** 1 Precision Medicine in Chronic Diseases Group, Respiratory Department, University Hospital Arnau de Vilanova and Santa María, Department of Nursing and Physiotherapy, Faculty of Nursing and Physiotherapy, University of Lleida, IRBLleida, 25198 Lleida, Spain; 2 Centro de Investigación Biomédica en Red de Enfermedades Respiratorias (CIBERES), Instituto de Salud Carlos III (ISCIII), 28029 Madrid, Spain; 3 Translational Research in Respiratory Medicine Group, Respiratory Department, University Hospital Arnau de Vilanova and Santa María, IRBLleida, 25198 Lleida, Spain; 4 Department of Experimental Medicine, University of Lleida-Biomedical Research Institute of Lleida (UdL-IRBLleida), 25198 Lleida, Spain; 5 Institut Català de la Salut, Atenció Primària, 25198 Lleida, Spain; 6 Research Support Unit Lleida, Fundació Institut Universitari per a la Recerca a l’Atenció Primària de Salut Jordi Gol i Gurina (IDIAPJGol), 08007 Lleida, Spain

**Keywords:** obstructive sleep apnea, blood pressure, non-dipping, metabolomic, lipidomic, metabolic pathways, CPAP

## Abstract

A non-dipping blood pressure (BP) pattern, which is frequently present in patients with obstructive sleep apnea (OSA), confers high cardiovascular risk. The mechanisms connecting these two conditions remain unclear. In the present study we performed a comprehensive analysis of the blood metabolipidome that aims to provide new insights into the molecular link between OSA and the dysregulation of circadian BP rhythmicity. This was an observational prospective longitudinal study involving adults with suspected OSA who were subjected to full polysomnography (PSG). Patients with an apnea–hypopnea index ≥ 5 events/h were included. Fasting plasma samples were obtained the morning after PSG. Based on the dipping ratio (DR; ratio of night/day BP values) measured via 24 h ambulatory BP monitoring, two groups were established: dippers (DR ≤ 0.9) and non-dippers (DR > 0.9). Treatment recommendations for OSA followed the clinical guidelines. Untargeted metabolomic and lipidomic analyses were performed in plasma samples via liquid chromatography–tandem mass spectrometry. Non-dipper patients represented 53.7% of the cohort (88/164 patients). A set of 31 metabolic species and 13 lipidic species were differentially detected between OSA patients who present a physiologic nocturnal BP decrease and those with abnormal BP dipping. Among the 44 differentially abundant plasma compounds, 25 were putatively identified, notably glycerophospholipids, glycolipids, sterols, and fatty acid derivates. Multivariate analysis defined a specific metabotype of non-dipping BP, which showed a significant dose-response relationship with PSG parameters of OSA severity, and with BP dipping changes after 6 months of OSA treatment with continuous positive airway pressure (CPAP). Bioinformatic analyses revealed that the identified metabolipidomic profile was found to be implicated in multiple systemic biological pathways, with potential physiopathologic implications for the circadian control of BP among individuals with OSA.

## 1. Introduction

Healthy sleep is closely linked to the physiological reduction in the sympathetic nervous system activity during the nighttime period, leading to a nocturnal decline in blood pressure (BP). Obstructive sleep apnea (OSA), the most prevalent sleep-disordered breathing, is a chronic disorder that stems from repetitive obstruction of the upper airway during sleep and affects nearly 1 billion people globally [1]. The cardinal features of OSA include intermittent hypoxia, sleep fragmentation, and swings in intrathoracic pressure [2]. Oxidative stress is regarded as a fundamental component of OSA pathophysiology, arising from an imbalance between the prooxidant and antioxidant systems [3]. The repetitive cycles of hypoxia and reoxygenation experienced in OSA are considered to be analogous to repeated ischemia–reperfusion injury [4], resulting in an increased production of reactive oxygen species (ROS) that exceeds the antioxidant supply [5,6]. Apneic episodes trigger hyperactivation of sympathetic nerve drive, resulting in transient vasoconstriction, accelerated heart rate, increased cardiac output, and surges in BP [7]. These acute hemodynamic disruptions, combined with the stress induced by intermittent hypoxia and frequent arousals from sleep, cause significant fluctuations in BP that manifest in a recurrent manner throughout the night. This situation can ultimately disrupt the circadian rhythmicity of arterial BP [8].

In the healthy state, a physiological decrease in BP occurs during sleep; this is referred to as a dipping pattern, and individuals with this pattern are designated dippers. Conversely, a reduction in or absence of this nocturnal BP decrease is termed a non-dipping pattern, and these individuals are classified as non-dippers [9]. A non-dipping BP pattern is widely recognized as a primary contributory risk factor for cardiovascular disease and organ damage [10,11,12,13,14,15,16,17,18]. Indeed, epidemiological investigations have reported that nighttime BP values are the strongest predictor of cardiovascular mortality [19]. Approximately 60% of individuals with OSA exhibit a non-dipping BP pattern [20]. The evidence linking OSA to impaired nocturnal dipping extends beyond cross-sectional data. Longitudinal studies have demonstrated that OSA serves as a risk factor for the development of this pathological BP pattern [21]. Moreover, the cooccurrence of OSA and a non-dipping BP pattern has been proposed to exert a multiplicative effect on adverse cardiovascular outcomes [22]. Unfortunately, neither the pathogenesis of OSA and the non-dipping pattern nor the interactions of these conditions have been completely elucidated [23]. A comprehensive understanding of the molecular mechanisms underpinning a non-dipping BP pattern in OSA may shed light on the physiopathological connection between these two prevalent conditions, and could help to design therapeutic interventions to prevent subsequent cardiometabolic damage.

Metabolomics, which is dedicated to the global study of all metabolites found in a given biospecimen, is one of the newest disciplines within the omics field [24]. The metabolome is defined as the complete collection of metabolites, i.e., small molecules (molecular mass < 15,000 Da), that are chemically transformed during cellular metabolism. Metabolites are the ultimate downstream products of multiple intracellular elements, including genes, transcriptional activators, RNA transcripts, protein transporters, enzymes, and other cellular components. Metabolomics is regarded as the final layer of the omics cascade, providing a direct functional readout of the biochemical activity of cells and tissues [25].

In chronic diseases such as OSA, the phenotype is complex and dynamic due to the interplay of multiple intrinsic and extrinsic factors. By incorporating the downstream input from the genome and the upstream input from environmental exposures, metabolomics can help to bridge the genotype-to-phenotype gap [26,27,28]. Global profiling of metabolites in accessible biosamples is currently being applied to investigate disease mechanisms and to identify new therapeutic targets in a variety of conditions [29,30,31]. This study presents a comprehensive analysis of the blood metabolipidome that aims to enhance our understanding of the molecular mechanisms underlying the alteration of nocturnal hemodynamic dipping in OSA.

## 2. Materials and Methods

### 2.1. Study Cohort

#### 2.1.1. Study Design

This was an observational, prospective, and longitudinal study involving consecutive adult participants referred to the sleep unit due to suspected OSA (ClinicalTrials.gov: NCT03513926, accessed on 1 August 2023). The exclusion criteria included the following situations: existence of a previously diagnosed sleep disorder, a history of OSA treatment, a psychophysical inability to complete the questionnaires, age > 60 years, or any medical, social, or geographical circumstance that, as determined by the responsible investigator, could affect the eligibility of the subject (e.g., pregnancy, drug or alcohol consumption, or life expectancy below 1 year). The Clinical Research Ethics Committee approved the study (University Hospital Arnau de Vilanova and Santa Maria of Lleida, No. 1153/1411), and all enrolled patients provided informed written consent for participation in the study.

#### 2.1.2. Baseline Clinical Evaluation

During the baseline visit, detailed information about sociodemographic characteristics, unhealthy lifestyle habits, and medical history, including comorbidities and prescribed medications, was gathered from the patients by trained clinicians. General physical and anthropometric parameters were documented, and the degree of self-reported daytime somnolence was assessed with the Epworth sleepiness scale (ESS) [32].

#### 2.1.3. Polysomnography for OSA Diagnosis

All patients who met the established selection criteria underwent an overnight in-lab polysomnography (PSG) sleep study using the Philips Sleepware G3 system (Amsterdam, Netherlands). All procedures were performed according to national guidelines and regulations for clinical practice [33]. The results from the sleep studies were analyzed by trained personnel using standard criteria [34]. Apnea was defined as a cessation or reduction in oronasal airflow of at least 90% for a minimum duration of 10 s. Hypopnea was defined as a 30–90% reduction in oronasal airflow for a minimum duration of 10 s, associated with oxygen desaturation ≥ 3% or evidence of arousal on the electroencephalogram. The apnea–hypopnea index (AHI) was computed based on the average number of apnea and hypopnea events/h of sleep. OSA severity parameters derived from PSG were evaluated according to international scoring guidelines [34], as previously published [35]. To ensure coverage of multiple OSA severity ranges, the analysis included patients with an AHI ≤ 5 events/h. To focus on the obstructive component of sleep apnea, patients with central apnea ≥ 50% were excluded from the analysis [36]. Treatment recommendations for the patients diagnosed with OSA were based on national clinical guidelines, according to usual clinical practice [37].

#### 2.1.4. Ambulatory BP Assessment

**Figure 1:**
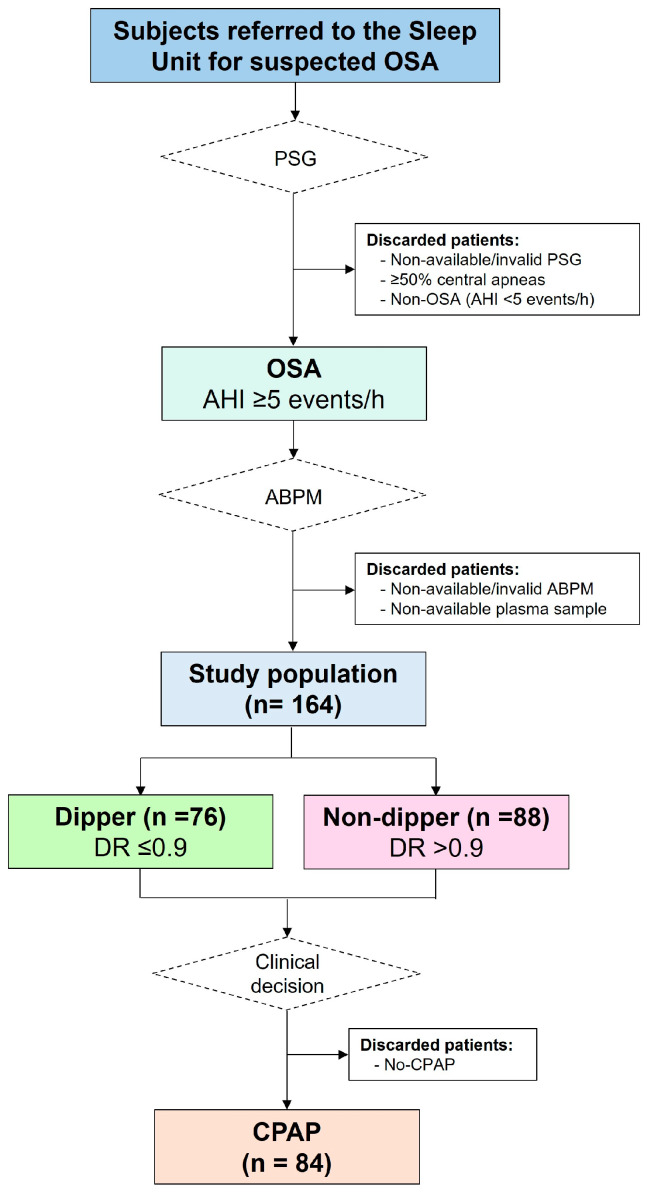
Flowchart of the study. Consecutive participants with suspected OSA who fulfilled the inclusion and exclusion criteria were subjected to in-laboratory PSG. Following ABPM, the patients were classified into two groups: dippers (DR ≤ 0.9) and non-dippers (DR > 0.9). Treatment recommendations for patients diagnosed with OSA were based on usual clinical practice, and patients treated with CPAP were evaluated after 6 months of follow-up. Abbreviations: ABPM: ambulatory blood pressure monitoring; AHI: apnea–hypopnea index; CPAP: continuous positive airway pressure; DR: dipping ratio; OSA: obstructive sleep apnea; PSG: polysomnography.

In the morning immediately after the sleep study, the patients were subjected to 24 h ambulatory BP monitoring (ABPM) (Mortara Ambulo 2400; Milwaukee, WI, USA), according to internationally recommended procedures [38]. Awake and asleep periods were defined using the sleeping time reported by each participant. BP measurements were obtained every 20 min during the daytime interval and every 30 min during the nighttime interval. ABPM recordings were considered optimal when >70% of the measurements were adequately recorded, with at least one measurement every hour; otherwise, the monitoring was repeated. The dipping ratios (DRs) for mean, systolic, and diastolic BPs were calculated as the ratios between the average nighttime and daytime BP values. Based on the DR of mean BP, the subjects were classified into two groups (Figure 1). A dipper pattern was defined as a nocturnal BP decrease of >10% relative to daytime values (DR ≤ 0.9), and a non-dipper pattern was defined as a nocturnal BP decrease of ≤10% relative to daytime values (DR > 0.9).

#### 2.1.5. Sample Collection

Overnight fasting venous blood samples were obtained at the same time of day (between 08:00 and 09:00 a.m.) the morning immediately after the sleep study. Whole-blood samples collected in ethylenediaminetetraacetic acid (EDTA) anticoagulant tubes (Vacuette, Greiner Bio-One, Kremsmünster, Austria) were centrifuged at 1500× *g* for 10 min at 4 °C to separate the plasma fraction. All specimens were immediately aliquoted, frozen, and stored in a dedicated −80 °C freezer. No freeze-thaw cycles were performed during the experiments.

#### 2.1.6. Post-Treatment Evaluation

Continuous positive airway pressure (CPAP) constitutes the first-line treatment for OSA. Patients undergoing CPAP treatment (according to national clinical guidelines [37]) were scheduled for a 6-month follow-up clinic visit. The follow-up visit included a physical examination and collection of information regarding current medication use, unhealthy life habits, and changes in anthropometric measurements. The level of adherence to the treatment protocol was recorded from the CPAP device. Good compliance was defined as the use of the CPAP device for an average of at least 4 h per night.

### 2.2. Metabolomic and Lipidomic Profiling

#### 2.2.1. Metabolite/Lipid Isolation and Untargeted Analysis

Metabolites and lipids were isolated from the same patient-derived plasma sample following previously validated extraction methods [39,40]. The metabolic and lipidic extracts were analyzed via liquid chromatography coupled to tandem mass spectrometry (LC-MS/MS), as previously described [41,42,43,44]. Ultra-high-performance liquid chromatography (UHPLC) was performed using an Agilent 1290 series system (Agilent Technologies, Santa Clara, CA, USA). Mass spectrometry analyses were performed via electrospray ionization quadrupole time of flight (ESI-Q-TOF) with an Agilent 6520 instrument (Agilent Technologies, Santa Clara, CA, USA). The order used to inject the samples was randomized, and quality control samples (pools of all the samples distributed in different aliquots and inserted after every five real samples) were used to control instrumental drift [45]. Data were acquired in both positive (+) and negative (−) ionization modes using MassHunter Data Acquisition software (Agilent Technologies, Barcelona, Spain) and were preprocessed using MassHunter Mass Profiler Professional software v15.1 (Agilent Technologies, Barcelona, Spain), as previously described. Data were normalized using a LOESS (LOcally WEighted Scatter-plot Smoother) signal correction approach [46]. To correct individual bias, only stable features (found in at least 70% of the quality control samples) were considered for the analyses [47].

#### 2.2.2. Feature Identification and Pathway Enrichment Analysis

According to previously published work, the potential identities of the features of interest, defined by exact molecular mass and retention time (RT), were compared against the Human Metabolome Database (HMDB) [48]. Potential identities were confirmed via comparison to the exact mass, RT, and MS/MS spectra fragmentation pattern of class-representative internal standards, when available, using data from public databases [49]. Bioinformatic pathway enrichment analyses were conducted using the MetaboAnalyst web service (https://www.metaboanalyst.ca/, accessed on 2 August 2023) [50]. Detailed information regarding the untargeted metabolomic and lipidomic analysis, feature identification, and pathway enrichment analysis can be found elsewhere [51].

### 2.3. Statistical Analysis

Descriptive statistics were used to determine the characteristics of the study population. The normality of the distributions was assessed by the Shapiro–Wilk test. The median [25th percentile; 75th percentile] and frequency (percentage) were used to summarize continuous and categorical data, respectively. The clinical and sociodemographic characteristics of the patients were compared between the dipper and non-dipper groups using the Mann-Whitney-Wilcoxon test for quantitative variables and the chi-squared test for categorical variables. Plasma metabolite and lipid levels were log-transformed for statistical purposes. Missing data were not imputed because there were no missing data for the variables used for the differential expression analysis. Linear models and empirical Bayes statistics were used to evaluate the differences in metabolite and lipid levels between the study groups [52], controlling for age, sex, body mass index (BMI), and antihypertensive medication use. Differential expression of metabolite/lipid species was defined as a significant difference (*p* value < 0.05) and a fold change (FC) > 1.15 (or <0.83 for downregulation) between the groups. Due to the exploratory nature of the study, *p* values were not adjusted for multiple comparisons. Linear dose-response relationships were assessed between the identified metabolite/lipid levels and the ABPM parameters using linear models adjusted for confounding factors. To evaluate the magnitudes of the identified associations, metabolite/lipid levels were standardized in this analysis.

A partial least squares-discriminant analysis (PLS-DA) was performed integrating the identified metabolite/lipid patterns previously associated with the baseline dipping status. Ten three-fold cross-validations were performed to select the optimal number of components. The types of associations between the selected components of PLS-DA with the DR as a continuous variable and the PSG parameters of OSA severity (AHI, TSat90 (time with oxygen saturation < 90%), and respiratory arousal index) were explored using generalized additive models (GAMs) with penalized thin plate regression splines. Similarly, we assessed the association of the first component of PLS-DA with the change in the DR after 6 months of treatment with CPAP. The threshold for statistical significance was set at a *p* value < 0.05 for all analyses. All statistical analyses were performed using R software, version 4.0.2 [53].

## 3. Results

### 3.1. Characteristics of the Study Groups at Baseline

A total of 164 OSA patients with available PSG data, ABPM data, and plasma sample were analyzed in this study. The population was middle-aged (median age 51 years) and overweight –obese (median BMI 29.3 kg/m^3^), and most participants were male (69.5%). The baseline characteristics of the study cohort according to nocturnal BP dipping category are outlined in Table 1. Non-dippers represented 53.7% of the cohort. As expected, marked differences were observed between the study groups in relation to ambulatory BP measurements. Differences in the DRs mainly stemmed from higher nighttime BP values within the non-dipper group. Non-dippers were more obese, presented a higher number of comorbidities, and therefore were more medicated. Compared to dippers, non-dippers exhibited increased values for the AHI and other OSA severity markers. Accordingly, individuals with a non-dipping profile exhibited lower sleep quality.

### 3.2. Untargeted Analysis of the Circulating Metabolipidome

**Figure 2:**
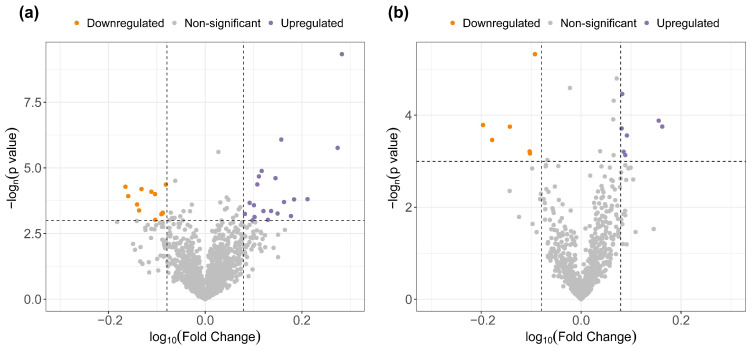
Untargeted plasma metabolomic (**a**) and lipidomic (**b**) profiling analysis. Volcano plots of the FC (*x*-axis) and *p* value (*y*-axis) for each detected metabolite and lipid in the comparison of non-dippers vs. dippers. Purple dots represent significantly downregulated (FC < 0.83) molecules, and orange dots represent significantly upregulated (FC > 1.25) molecules in non-dipper patients. The results are adjusted for confounding factors (age, sex, BMI, and antihypertensive medications). The threshold used to define statistical significance was *p* value < 0.05. Abbreviations: BMI: body mass index; FC: fold change.

The first objective of the study was to assess the differences in the blood metabolome and lipidome between OSA patients who present a physiologic nocturnal BP decrease and those with abnormal BP dipping. An untargeted metabolomic and lipidomic approach was applied via LC-MS/MS, the gold-standard technique for this purpose. After quality control and signal correction, 1506 metabolites and 748 lipids were detected in the plasma and included in the unsupervised analysis. After adjustment for confounding factors, 31 metabolic species and 13 lipidic species were found to be differentially expressed between dipper and non-dipper OSA patients (Figure 2 and Supplementary Tables S1 and S2). Out of the 44 differentially expressed metabolites and lipids, a total of 25 compounds were putatively annotated based on the physicochemical properties and/or spectral similarity with public/commercial spectral libraries (Table 2). Collectively, the identified features of interest were mainly lipidic species, with a smaller set of lysine derivatives. The most affected lipid classes were glycerophospholipids (lysophospholipids, phosphatidic acid, cardiolipin, phosphatidyl-choline, and serine), glycerolipids (diacylglycerols), sterols (bile acids and steroids), and fatty acid derivatives. These 25 putative features of interest were included in the subsequent analyses.

### 3.3. Plasma Metabotype Associated with Impaired BP Dipping in OSA

**Figure 3:**
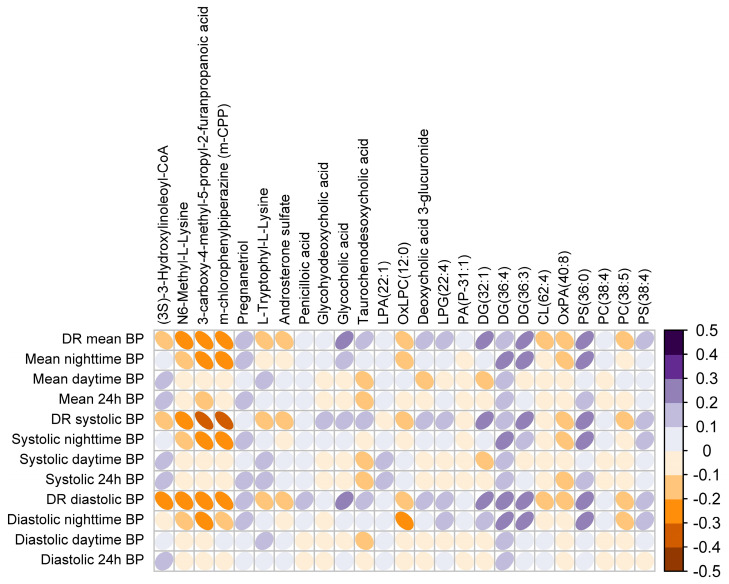
Adjusted linear associations between ABPM parameters and the identified metabolites and lipids. The color scale illustrates the degree of correlation and ranges from orange to purple, indicating negative to positive correlations, respectively. The results are adjusted for confounding factors (age, sex, BMI, and antihypertensive medications). Abbreviations: ABPM: ambulatory blood pressure monitoring; BP: blood pressure; CL: cardiolipin; DG: diacylglycerol; DR: dipping ratio; PG: phosphatidylglycerol; PA: phosphatidic acid; PC: phosphatidylcholine; PS: phosphatidylserine.

Once we had established the differentially expressed metabolites and lipids in plasma based on dipping status, we sought to evaluate the linear association of this circulating profile with ambulatory measures of BP. As depicted in Figure 3, the identified molecular profile was broadly associated with ABPM variables, not only with the DR of mean BP but also with the DRs of systolic and diastolic BP and especially with nighttime BP values.

**Figure 4:**
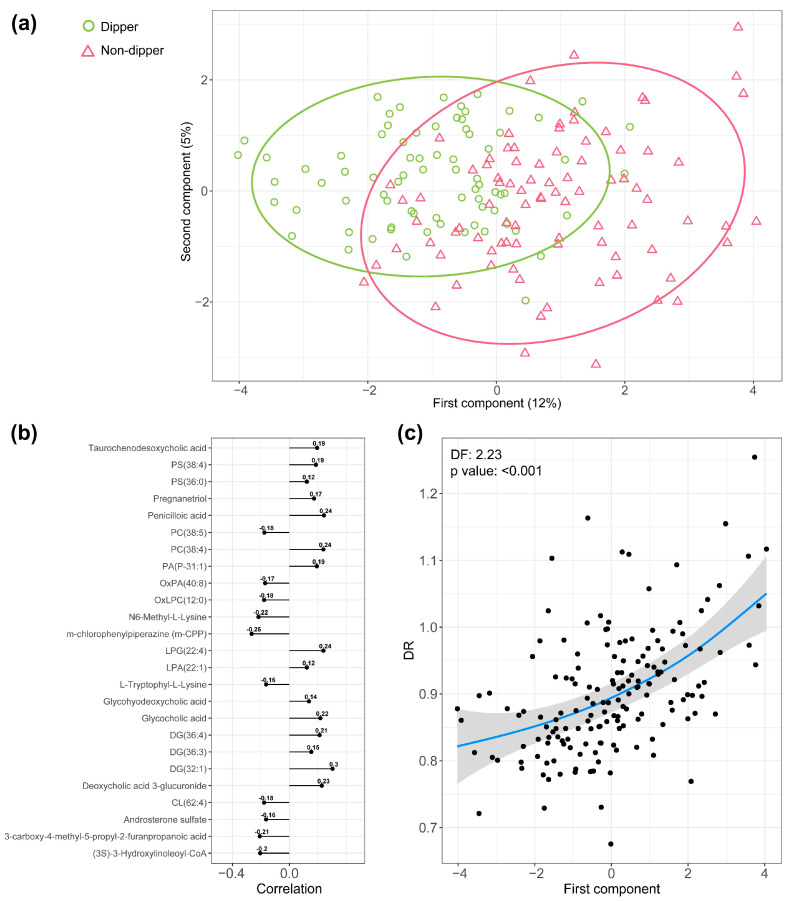
Plasma metabotype associated with impaired BP dipping in OSA. (**a**) PLS-DA discriminating between OSA patients with a dipping BP pattern and OSA patients with a non-dipping BP pattern. Each point represents a patient. Dipper patients appear in green, and non-dipper patients appear in pink. (**b**) Loading of each identified metabolite and lipid to the first component of PLS-DA. (**c**) GAMs with penalized thin plate regression splines representing the first component of PLS-DA (*x*-axis) and the DR as a continuous variable (*y*-axis). Each point represents a patient. The blue line represents the estimate of the change in DR according to the first component of PLS-DA, and the grey area corresponds to the 95% confidence interval. The results are adjusted for confounding factors (age, sex, BMI, and antihypertensive medications). Abbreviations: BMI: body mass index; BP: blood pressure; CL: cardiolipin; DF: estimated degrees of freedom; DG: diacylglycerol; GAM: generalized additive model; OSA: obstructive sleep apnea; PA: phosphatidic acid; PC: phosphatidylcholine; PG: phosphatidylglycerol; PLS-DA: partial least-squares discriminant analysis; PS: phosphatidylserine.

A multivariate analysis based on PLS-DA was applied to the differentially detected metabolites and lipids. Figure 4a shows that the plasma metabolipidomic levels were able to separate patients with a dipping profile from those with a non-dipping profile, depicting a differential plasma metabotype associated with impaired BP dipping. Given that the first component of PLS-DA was the key contributor for separating the study groups (non-dippers vs. dippers), we performed an in-depth study on the loading of each feature to this component (Figure 4b). As illustrated in Figure 4c, the identified metabotype was significantly associated with the DR as a continuous variable.

We then explored the association of the metabolipidomic profile generated by PLS-DA with OSA severity markers, including the AHI as the primary disease-defining metric, the TSat90 as a hallmark of nocturnal hypoxemia, and the respiratory arousal index as a hallmark of sleep fragmentation. A positive dose-response relationship was found between the parameters of OSA severity and the plasma metabolipidomic signature (Figure S1).

### 3.4. Association of the Metabolipidomic Fingerprint with Changes in the DR after OSA Treatment with CPAP

Treatment recommendations for patients diagnosed with OSA were based on usual clinical practice. A total of 84 patients were treated with CPAP and evaluated after 6 months of therapy. The median [p_25_; p_75_] of the CPAP use value was 4.87 [3.67; 6.42], with good compiler patients (average use ≥4 h/day) representing 68.3% of all treated patients. The changes in the ABPM parameters between the beginning of the study and the posttreatment period are presented in Table S3. In brief, slight reductions in all ABPM variables (24 h BPs, daytime BPs, and nighttime BPs) were observed. The observed reduction in the mean nocturnal BP after CPAP treatment was −3.49 mmHg (95% CI: [−6.30; −0.68]). The variation in the DR was 0.04 (95% CI: [0.00; 0.08]) for patients who presented a dipping pattern at baseline and −0.06 (95% CI: [−0.09; −0.03]) for those who presented a non-dipper profile at baseline.

**Figure 5:**
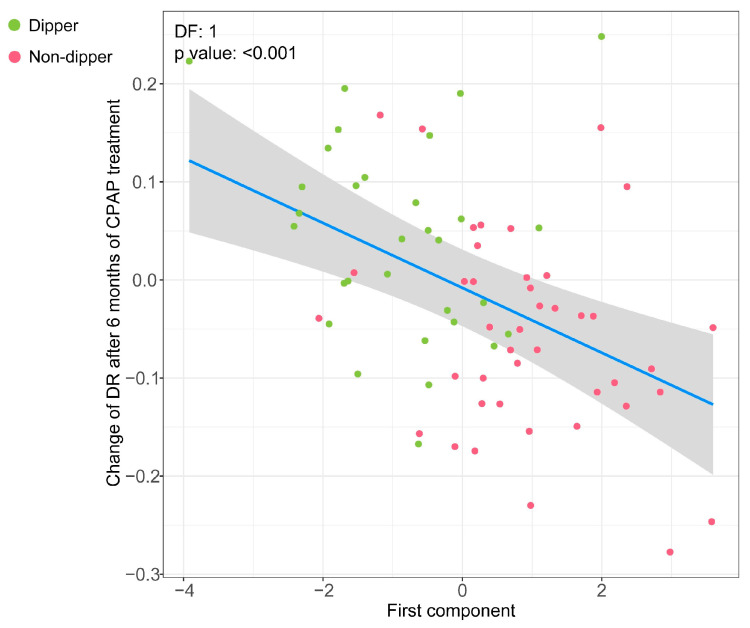
Association of the baseline metabolipidomic signature of impaired BP dipping with the variation in the DR after 6 months of OSA treatment with CPAP. GAMs with penalized thin plate regression splines illustrating the first component of PLS-DA (*x*-axis) and the change in the DR after CPAP treatment (*y*-axis). The blue line represents the estimate of the change in the post-treatment DR according to the first component of PLS-DA, and the grey area corresponds to the 95% confidence interval. Each point represents a patient. Patients with a dipping pattern at baseline appear in green, and those with a baseline non-dipping pattern appear in pink. The results are adjusted for confounding factors (age, sex, BMI, and antihypertensive medication use). Abbreviations: BMI: body mass index; BP: blood pressure; CPAP: continuous positive airway pressure; DF: estimated degrees of freedom; GAM: generalized additive model; OSA: obstructive sleep apnea.

We next tested whether the baseline metabolipidomic signature of impaired BP dipping was able to explain the modulation in the DR after 6 months of OSA treatment with CPAP. As displayed in Figure 5, the molecular profile identified at baseline was linearly associated with changes in the DR after CPAP use (Figure 5). Higher loadings of the first component were associated with a post-treatment decrease in the DR (mainly representing non-dipper patients), whereas lower loadings of the first component at baseline were related to an increase in the DR after CPAP treatment (primarily representing non-dipper patients).

### 3.5. Pathway Enrichment Analysis

**Figure 6:**
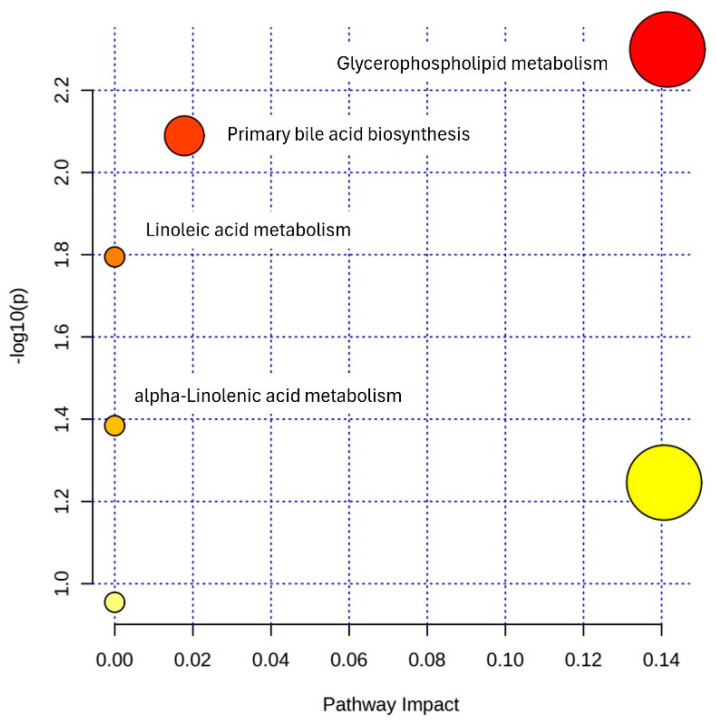
Pathway enrichment analysis including the metabolipidomic features identified as relevant to circadian BP control in OSA. Scatter plot presenting the enriched metabolic pathways in which the identified metabolites and lipids may be involved. Each circle represents a pathway. The color gradient indicates the significance of the pathway according to *p* value, with yellow indicating higher *p* values and red indicating lower *p* values (*y*-axis). The size of the circle represents the impact score of the pathway based on the number of molecules contained in the pathway (*x*-axis). Significantly affected pathways with a *p* value < 0.05 appear with their name.

Finally, to gain an understanding of the molecular mechanisms in which the identified metabolipidomic profile may be involved, we performed a bioinformatic in silico analysis using the online software MetaboAnalyst v4.0. Four enriched pathways, namely, glycerophospholipid metabolism, primary bile acid biosynthesis, linoleic acid metabolism, and alpha-linolenic acid metabolism, met the criteria at the defined significance level (Figure 6). Additionally, a number of metabolite sets involved in multiple systemic metabolic pathways were found to be significantly enriched in the bioinformatic analysis (Figure S2).

## 4. Discussion

This study comprehensively explores the blood metabolomic and lipidomic landscape underlying the circadian variation in BP in patients with OSA. We report the following findings: (i) a set of plasma metabolites and lipids were differentially detected in OSA patients with abnormal nocturnal BP dipping; (ii) the circulating levels of this profile were linearly associated with ambulatory measures of BP; (iii) multivariate analysis defined a circulating metabotype associated with impaired BP dipping, which was highly correlated with OSA severity measures; (iv) the identified baseline molecular signature showed a dose-response relation with the modulation of BP dipping after OSA treatment with CPAP; and (v) the metabolipidomic profile, which was mainly composed of lipidic species, was implicated in multiple systemic biological pathways, notably in the metabolism of glycerophospholipids and bile acids.

Apnea and hypopnea, along with the consequent compensatory hyperpnea, are associated with decreased parasympathetic and increased sympathetic activity [54]. Elevated sympathetic outflow, most notably occurring at the end of respiratory events, antagonizes the natural BP dipping phenomenon, causing surges in intravascular pressure. The loss or disruption of nocturnal hemodynamic dipping causes important adverse health consequences. Nevertheless, there is a limited understanding of the molecular components connecting OSA to the disruption of circadian BP rhythmicity.

Biofluids provide a close representation of the metabolic activity of the organs from which they are derived or the organs they bathe. Since blood irrigates all organs and tissues, blood-based profiling analyses can serve as a reliable metabolic proxy for the entire organism [31]. Untargeted metabolomics analysis has demonstrated wide-ranging applicability in revealing unanticipated metabolic changes across various biological conditions due to its sensitivity, high-throughput capabilities, and minimal sample needs. Although small in number, some studies using metabolomic and lipidomic approaches have been conducted to evaluate biomarkers and explore the physiopathological mechanisms of BP and hypertension [55,56,57,58]. However, to date, no study has specifically addressed the impact of the 24-h circadian variation in BP on the circulating metabolipidome.

A metabotype, or metabolic phenotype, can be defined as a group of individuals characterized by similarities in metabolic profiles [26], which in turn result from interactions among lifestyle, the gut microbiome, genetics, and environmental factors. Here, we identified a circulating metabotype associated with impaired BP dipping in OSA, which was associated with OSA severity markers and BP dipping changes after OSA treatment. Among the two main hallmarks of OSA, namely, nocturnal hypoxemia and sleep fragmentation, we found that the latter showed the strongest correlation with the identified metabotype. This finding aligns with our previous research, where a comprehensive characterization of the polysomnographic determinants of non-dipping in OSA patients revealed that the respiratory arousal index was a key parameter related to the loss of nocturnal dipping [35]. Recurrent arousals from sleep could have a significant impact on the levels of the metabolites and lipids that are detectable in the peripheral blood. Our results indicate that sleep fragmentation represents a relevant mechanistic pathway underpinning the link between OSA and the dysregulation of BP rhythmicity, which may be reflected in a specific plasma metabotype.

The specific blood-based metabotype identified here primarily involved glycerophospholipids (10 out of 25), sterols (6), glycerolipids (3), fatty acids (2), and amino acids/peptides (2). This suggests that the metabolic impact of a non-dipping BP profile in OSA would be mainly manifested as dysregulation of phospholipid, sterol, and fatty acid metabolism. Globally, three functional categories associated with the different lipid classes could be inferred: metabolic intermediates, bioenergetic compounds, and bioactive lipids. Importantly, most of these lipid species could be involved in the circadian regulation of BP rhythms.

Among glycerolipids, diacylglycerols represent the main subclass of lipids associated with the non-dipper condition in OSA. Vasopressins are known to bind G-protein coupled receptors and activate intracellular phospholipase-C [59], generating diacylglycerol as an end-product of the reaction, which, along with the secondary messenger inositol-1,4,5-triphosphate, regulates the cytosolic concentration of calcium ions and protein kinase C (PKC) activity. Elevated PKC activity is known to promote oxidative stress through the stimulation of ROS-producing enzymes [60,61], and to increase expression of inflammatory factors [62], which can exacerbate the cellular oxidative environment and contribute to endothelial dysfunction in OSA. Indeed, vascular endothelial inflammation and enhanced endothelial oxidative stress provide a starting point to elucidate the mechanisms mediating the association between OSA and BP impairments [63]. Increased expression of these lipid classes in non-dippers suggests an upregulation of the diacylglycerol axis. Therefore, it is biologically plausible to postulate a pathophysiological role for diacylglycerols in the regulation of nocturnal BP [64], although evidence demonstrating a direct relation between the plasma content of diacylglycerols and the activation of intracellular signaling pathways involving diacylglycerols is currently lacking. Additionally, in line with our findings, DG(36:3) has been previously described as a cross-species lipid marker of sleep restriction and sleep duration [65], reinforcing the role of OSA in the observed associations.

Glycerophospholipids are the class of lipids that have been shown to be the most affected by the non-dipping pattern in OSA, with 10 lipid species found to be differentially expressed compared to dipper patients: lysophospholipids (3), phosphatidic acid (2), phosphatidylcholine (2), phosphatidylserine (2), and cardiolipin (1). All these glycerophospholipid subclasses, along with diacylglycerols, exhibit a strongly interdependent relationship, either as precursors or products, in their biosynthesis pathways [66], suggesting a potential alteration in phospholipid biosynthesis in patients with absent nocturnal BP dipping. Interestingly, the phosphatidylcholine and serine forms, as well as cardiolipin, have been demonstrated to have biosynthetic, structural, and functional links with mitochondria [67]. Alterations in the expression of these lipids could be the result of an alteration in the bioenergetic capacity of the affected cell types, with subsequent effects on BP regulation. Reinforcing this idea, lower levels of an acyl-coenzyme A, which is the form in which fatty acids are used in mitochondrial beta-oxidation, were detected in non-dippers. Mitochondrial dysfunction is a major source of ROS production [68]. ROS can act as signaling molecules that play important regulatory roles in a plethora of physiological processes [69], potentially including the circadian regulation of BP. Two additional considerations should be noted. First, the affected phosphatidylcholine and serine species are characterized by four and five double bonds. This suggests the presence in their structure of 20:4 (arachidonic acid) and 20:5 (eicosapentaenoic acid) fatty acids, which are both precursors for the biosynthesis pathways of eicosanoids, which, among several functions, are known to play a role in vasodilatation and BP fluctuation [70,71]. The second is the involvement of various lysophospholipids and phosphatidic acids, which, in addition to being intermediates in the biosynthesis of glycerophospholipid precursors, are also known to be bioactive lipids that may also participate in the regulation of BP [72]. 

Sterols are another relevant class of lipids that were differentially detected in non-dipper patients. Six differentially abundant sterols were identified: two were derivative metabolites of sex hormones, and the four remaining sterols were bile acids and conjugates. Available evidence suggests that these molecules could affect BP control, acting as bioactive lipids. In this sense, bile acids are known to be able to reduce BP by attenuating vascular reactivity [73], while conjugated bile acids are known to be inversely correlated with BP in humans and rats [74]. Furthermore, bile acids may function as bioactive lipids that activate different nuclear receptors [75] regulating hypoxia inducible factor-1-alpha (HIF-1-alpha) under hypoxic conditions [76], suggesting the activation of adaptive mechanisms directed to counter OSA-derived injury. Additionally, some forms of bile acids have been associated with reduced oxidative stress and decreased inflammation in many in vitro and in vivo models of various diseases, mostly due to a cytoprotective effect. The mechanisms underlying this cytoprotective activity have been mainly attributed to alleviation of endoplasmic reticulum stress and stabilization of the unfolded protein response [77]. Although the underlying molecular mechanisms remain unknown, our findings and observations from other authors suggest a potential adaptative protective role for bile acid metabolism in the alteration of BP regulation in OSA patients.

Overall, the metabolipidomic signature identified in this study could hold potential implications in the circadian control of BP, and may represent the first steps to establish the molecular bases underlying the dysregulation of nocturnal BP control, frequently observed in OSA patients. Additionally, the metabolipidomic signature identified in this study could potentially constitute a source of therapeutic targets. 

Our study has certain limitations that should be noted. First, due to the observational design of the study, cause-effect relationships remain unproven. Second, although we adjusted the analyses for various confounding factors, individual biologic conditions and/or lifestyle behaviors, such as shift work routines, diet, caffeine intake, psychological stress, or antihypertensive drug intake timing, may have impacted the observed associations. Third, due to the incipient state of this research field, an exploratory and unbiased methodological approach was applied, aiming to gain new insights from which new hypotheses might be developed. Therefore, further studies are needed to determine the validity of our findings in another independent group of individuals.

The strengths of this study include the evaluation of a relatively large cohort of consecutive participants who were referred to the sleep unit for suspected OSA. This resulted in a realistic degree of heterogeneity within the OSA study population and facilitated the evaluation of the entire spectrum of disease severity. Other strengths include the use of 24-h ABPM and PSG, which are gold-standard techniques for identifying the circadian patterns of BP and diagnosing OSA, respectively. In addition, the ABPM and PSG data enabled us to explore the relationships among BP variables, OSA variables, and circulating metabolomic and lipidomic levels. This approach mitigates the potential for identifying misleading or non-representative associations based solely on dipping status and OSA status. Finally, the longitudinal design of the study and the consequent evaluation of the relation between the metabolipidomic signature and the effect of CPAP treatment on the evolution of the DR reinforce the etiological role of OSA in the observed associations.

## 5. Conclusions

To our knowledge, this study is the first to apply a systemic metabolipidomic profiling strategy to enhance our understanding of the molecular mechanisms underlying the alteration of nocturnal hemodynamic dipping in OSA. We identified a specific plasma metabotype of impaired BP dipping, which was associated with polysomnographic parameters of OSA severity and BP dipping variations after OSA treatment with CPAP. The identified metabotype was primarily composed of lipidic species, notably glycerophospholipids, sterols, and glycerolipids. The affected lipidic classes could have dual cytoprotective/physiopathologic implications for the circadian control of BP rhythmicity, through oxidative stress-related pathways. This exploratory study provides hypothesis-generating data and may form the basis for future investigations. However, further research involving additional cohorts and functional analyses are needed to corroborate these findings.

## Figures and Tables

**Table 1:** Clinical, ABPM, and PSG characteristics of the study population at baseline. Data are presented as the median [p_25_; p_75_] for quantitative variables and n (%) for qualitative variables. *p* values < 0.05 are presented in bold. Abbreviations: ABPM: ambulatory blood pressure monitoring; ACE: angiotensin-converting enzyme; AHI: apnea–hypopnea index; BMI: body mass index; BP: blood pressure; DR: dipping ratio; ESS: Epworth sleepiness scale; OSA: obstructive sleep apnea; R: rapid eye movement; SaO_2_: oxygen saturation; TSat90: time with SaO_2_ < 90%.

	All N = 164	Dippers N = 76	Non-Dippers N = 88	*p* Value
**Clinical data**				
** Demographic/anthropometric**				
Age (years)	51.0 [44.8; 55.0]	50.0 [44.8; 56.0]	52.0 [44.8; 55.0]	0.504
Sex				0.112
Male	114 (69.5%)	58 (76.3%)	56 (63.6%)	
Female	50 (30.5%)	18 (23.7%)	32 (36.4%)	
BMI (kg/m^2^)	29.3 [26.7; 33.4]	28.5 [26.1; 31.8]	30.2 [27.1; 34.6]	**0.029**
** Smoking status**				
Never	67 (40.9%)	27 (35.5%)	40 (45.5%)	0.433
Former	52 (31.7%)	26 (34.2%)	26 (29.5%)	
Current	45 (27.4%)	23 (30.3%)	22 (25.0%)	
** Comorbidities**				
Diabetes	17 (10.4%)	4 (5.26%)	13 (14.8%)	0.083
Hypertension	56 (34.1%)	21 (27.6%)	35 (39.8%)	0.142
Dyslipidemia	38 (23.5%)	12 (16.2%)	26 (29.5%)	0.071
Cardiovascular disease	29 (17.7%)	9 (11.8%)	20 (22.7%)	0.106
** Medication use**				
Insulin	7 (4.27%)	1 (1.32%)	6 (6.82%)	0.124
Any antihypertensive drug	62 (37.8%)	21 (27.6%)	41 (46.6%)	**0.020**
ACE inhibitors	41 (25.0%)	13 (17.1%)	28 (31.8%)	**0.047**
Beta-blockers	29 (17.7%)	12 (15.8%)	17 (19.3%)	0.700
Diuretic agents	23 (14.1%)	6 (8.00%)	17 (19.3%)	0.065
Calcium-channel blockers	14 (8.59%)	4 (5.26%)	10 (11.5%)	0.256
Angiotensin II receptor blockers	13 (8.02%)	3 (3.95%)	10 (11.6%)	0.132
Lipid-lowering drugs	32 (19.6%)	8 (10.5%)	24 (27.6%)	**0.011**
**ABPM data**				
** Dipping ratios**				
24 h DR	0.91 [0.85; 0.97]	0.85 [0.82; 0.87]	0.97 [0.93; 1.00]	**<0.001**
Systolic DR	0.92 [0.86; 0.98]	0.86 [0.83; 0.89]	0.96 [0.93; 1.02]	**<0.001**
Diastolic DR	0.91 [0.87; 0.97]	0.86 [0.84; 0.89]	0.96 [0.92; 1.00]	**<0.001**
** Nighttime BP**				
Mean (mmHg)	88.8 [80.3; 97.5]	83.2 [77.4; 91.3]	94.0 [85.3; 106]	**<0.001**
Systolic (mmHg)	118 [106; 136]	110 [103; 127]	122 [112; 142]	**<0.001**
Diastolic (mmHg)	74.6 [68.9; 79.0]	70.4 [67.0; 75.4]	76.6 [72.8; 84.4]	**<0.001**
** Daytime BP**				
Mean (mmHg)	97.1 [90.5; 106]	99.0 [92.3; 108]	96.0 [89.0; 103]	**0.034**
Systolic (mmHg)	128 [120; 143]	128 [121; 145]	127 [119; 141]	0.292
Diastolic (mmHg)	81.3 [76.2; 85.4]	82.1 [77.1; 87.2]	79.6 [74.7; 84.8]	**0.029**
** 24 h BP**				
Mean (mmHg)	95.0 [88.5; 105]	94.6 [89.1; 105]	95.8 [88.1; 104]	0.924
Systolic (mmHg)	125 [117; 140]	123 [117; 139]	128 [117; 142]	0.498
Diastolic (mmHg)	79.4 [74.0; 84.2]	79.4 [74.8; 84.4]	79.4 [73.5; 84.2]	0.568
**Polysomnography data**				
** Respiratory disturbances**				
AHI (events/h)	28.6 [14.0; 50.4]	22.4 [11.8; 41.8]	34.9 [21.4; 60.5]	**0.001**
Obstructive apnea index (events/h)	5.23 [1.59; 14.5]	3.26 [1.30; 12.4]	6.46 [1.92; 26.2]	**0.043**
Hypopnea index (events/h)	18.0 [9.89; 27.2]	13.0 [8.50; 25.1]	20.8 [12.0; 30.4]	**0.011**
** Nocturnal hypoxemia**				
Mean SaO_2_ (%)	94.0 [92.0; 95.0]	94.0 [93.0; 95.0]	93.0 [91.0; 95.0]	0.052
Minimum SaO_2_ (%)	83.0 [76.0; 88.0]	85.0 [78.2; 89.0]	82.0 [73.5; 87.0]	**0.027**
TSat90 (%)	1.97 [0.22; 8.30]	1.33 [0.16; 4.50]	2.40 [0.29; 15.9]	**0.028**
Desaturation index (events/h)	4.47 [1.27; 19.4]	4.34 [1.19; 10.4]	7.01 [1.46; 23.8]	0.268
** Sleep fragmentation**				
Respiratory arousal index (events/h)	20.1 [8.92; 37.2]	15.0 [7.14; 24.2]	25.8 [14.5; 46.8]	**<0.001**
Movement arousal index (events/h)	3.49 [1.33; 6.59]	3.67 [1.71; 5.50]	3.49 [1.31; 7.42]	0.564
Unspecific arousal index (events/h)	6.32 [3.04; 10.6]	6.46 [2.61; 9.90]	6.21 [3.19; 11.9]	0.927
** Sleep architecture**				
Stage N1 (%)	11.4 [7.09; 16.9]	11.3 [6.50; 16.8]	11.8 [7.44; 17.2]	0.737
Stage N2 (%)	43.7 [37.6; 53.2]	47.0 [40.5; 53.8]	40.4 [34.2; 52.1]	**0.014**
Stage N3 (%)	26.3 [17.6; 37.2]	24.2 [15.9; 35.2]	27.8 [19.1; 38.9]	0.130
Stage R (%)	14.1 [9.59; 18.3]	14.1 [11.0; 17.2]	14.1 [9.00; 19.3]	0.992
** Sleep quality**				
Total sleep time (min)	346 [318; 375]	344 [322; 372]	347 [311; 376]	0.786
Sleep latency (min)	14.6 [7.80; 29.0]	12.5 [7.03; 20.1]	18.0 [8.00; 32.4]	0.085
Sleep efficiency (%)	86.0 [77.9; 90.6]	87.2 [79.9; 91.7]	83.2 [76.9; 89.4]	**0.025**
Total wake time (min)	58.0 [36.2; 89.7]	51.4 [30.2; 84.0]	68.1 [48.0; 96.0]	**0.021**
WASO (min)	40.0 [24.5; 68.7]	35.7 [21.3; 52.0]	47.8 [29.3; 72.5]	0.062
Somnolence (ESS)	11.0 [7.00; 14.5]	11.0 [7.00; 14.0]	11.0 [7.00; 15.0]	0.736

**Table 2:** Potential identities of the significantly differentially expressed metabolites and lipids between non-dipper and dipper OSA patients. The methodological approach used to detect the molecules is represented as M for metabolomics and L for lipidomics. All compounds were putatively annotated based on physicochemical properties and/or spectral similarity with public/commercial spectral libraries: (a) ID based on exact mass, RT, and MS/MS spectrum; (b) ID based on exact mass and RT. Abbreviations: CL: cardiolipin; DG: diacylglycerol; PG: phosphatidylglycerol; PA: phosphatidic acid; PC: phosphatidylcholine; PS: phosphatidylserine; RT: retention time.

Mass	RT (min)	Method	Regulation Dipper vs. Non-Dipper	Putative Identification	Class	Reliability
791.6017	11.42984	M	Up	PC(38:4)	GP	b
598.2597	8.905328	M	Up	LysoPG(22:4)	GP	b
791.5651	10.62096	M	Up	PS(36:0)	GP	b
598.4338	11.2998	M	Up	PA(P-31:1)	GP	b
811.5371	14.02962	M	Up	PS(38:4)	GP	a
514.31245	10.82806	M	Up	LysoPA(22:1)	GP	a
760.4527	11.71748	M	Down	OxPA(40:8)	GP	b
658.4229	11.58019	M	Down	CL(62:4)	GP	a
515.2295	11.71635	M	Down	OxLysoPC(12:0)	GP	b
807.5784	7.231465	L	Down	PC(38:5)	GP	a
465.3101	8.988933	M	Up	Glycocholic acid	ST	a
568.3252	9.260691	M	Up	Deoxycholic acid 3-glucuronide	ST	b
449.3152	8.721428	M	Up	Glycohyodeoxycholic acid	ST	b
499.2975	10.32784	M	Up	Taurochenodesoxycholic acid	ST	a
318.26	11.57911	M	Up	Pregnanetriol	ST	b
370.1825	8.089659	M	Down	Androsterone sulfate	ST	a
602.4646	11.71269	M	Up	DG(32:1)	GL	b
616.5038	8.262866	L	Up	DG(36:4)	GL	b
635.5499	7.994549	L	Up	DG(36:3)	GL	a
240.1008	7.275254	M	Down	3-carboxy-4-methyl-5-propyl-2-furanpropanoic acid	FA	b
1079.255	7.688919	L	Down	(3S)-3-Hydroxylinoleoyl-CoA	FA	b
160.1219	0.5020314	M	Down	N6-Methyl-L-Lysine	AA	a
368.1674	8.3971	M	Down	L-Tryptophyl-L-Lysine	AA	a
412.1364	11.15013	M	Up	Penicilloic acid	Drug	a
256.0955	4.755068	M	Down	m-chlorophenylpiperazine (m-CPP)	Drug	b
827.1027	0.5837527	M	Up	Unknown		
822.2788	11.38875	M	Up	Unknown		
816.2971	11.38298	M	Up	Unknown		
1108.853	11.57688	M	Up	Unknown		
764.4844	12.07795	M	Up	Unknown		
1212.914	11.70673	M	Up	Unknown		
1249.356	9.835346	L	Up	Unknown		
1338.199	10.34988	L	Up	Unknown		
1171.264	7.82933	L	Up	Unknown		
1266.198	10.65473	L	Up	Unknown		
1384.454	9.951343	L	Up	Unknown		
474.2918	12.98659	M	Down	Unknown		
606.0906	12.08467	M	Down	Unknown		
1025.551	13.29278	M	Down	Unknown		
542.1265	8.394325	M	Down	Unknown		
166.9866	0.9055392	L	Down	Unknown		
665.0966	5.214503	L	Down	Unknown		
1428.382	10.15159	L	Down	Unknown		
305.3186	2.950193	L	Down	Unknown		

## Data Availability

The data presented in this study are available on request from the corresponding author.

## Supplementary Materials

The following supporting information can be downloaded at: https://www.mdpi.com/article/10.3390/antiox12122047/s1. Figure S1: Association of the plasma metabolipidomic signature of impaired BP dipping with OSA severity parameters. Figure S2: Metabolite set enrichment analysis including the metabolipidomic features identified as relevant to circadian BP control in OSA. Table S1: Differentially expressed features between non-dipper and dipper patients in the metabolomic analysis. Table S2: Differentially expressed features between non-dipper and dipper patients in the lipidomic analysis. Table S3. Changes in the ABPM parameters after 6 months of OSA treatment with CPAP.
