# Supplementary material for: Metabolipidomic Analysis in Patients with Obstructive Sleep Apnea Discloses a Circulating Metabotype of Non-Dipping Blood Pressure
Source: Antioxidants (Basel). 2023 Nov 27;12(12):2047. doi: 10.3390/antiox12122047 (PMC10741016; doi:10.3390/antiox12122047)
Supplement: Supplementary file 1 [file antioxidants-12-02047-s001.zip › antioxidants-2656946-supplementary.pdf]

SUPPLEMENTARY INFORMATION

Metabolipidomic Analysis in Patients with Obstructive Sleep Apnea Discloses a Circulating Metabotype of Non-Dipping Blood Pressure

Lucía Pinilla [1,2], Iván D. Benítez [2,3], Esther Gracia-Lavedan [2,3], Gerard Torres [2,3], Olga Mínguez [3], Rafaela Vaca [3], Mariona Jové [4], Joaquim Sol [4,5,6], Reinald Pamplona [4], Ferran Barbé [2,3], Manuel Sánchez-de-la-Torre [1,2] *

1 Precision Medicine in Chronic Diseases Group, Respiratory Department, University Hospital Arnau de Vilanova and Santa María; Department of Nursing and Physiotherapy, Faculty of Nursing and Physiotherapy, University of Lleida, IRBLleida, Lleida, Spain.
2 Centro de Investigación Biomédica en Red de Enfermedades Respiratorias (CIBERES), Instituto de Salud Carlos III (ISCIII), Madrid, Spain.
3 Translational Research in Respiratory Medicine Group, Respiratory Department, University Hospital Arnau de Vilanova and Santa María, IRB Lleida, Lleida, Spain.
4 Department of Experimental Medicine, University of Lleida-Biomedical Research Institute of Lleida (UdL-IRBLleida), Lleida, Spain.
5 Institut Català de la Salut, Atenció Primària, Lleida, Spain.
6 Research Support Unit Lleida, Fundació Institut Universitari per a la Recerca a l'Atenció Primària de Salut Jordi Gol i Gurina (IDIAPJGol), Lleida, Spain.

* Correspondence: Manuel Sánchez-de-la-Torre. sanchezdelatorre@gmail.com. Avinguda Rovira Roure 80, E-25198 Lleida, Spain.

TABLE OF CONTENTS

Figure S1 – Page 3

Figure S2 – Page 4

Table S1 – Page 5

Table S2 – Page 6

Table S3 – Page 7

Figure S1. Association of the plasma metabolipidomic signature of impaired BP dipping with OSA severity parameters. GAM models with penalized thin plate regression splines illustrating the first component (x-axis) and the OSA severity parameters (y-axis): (a) AHI, (b) TSat90, and (c) respiratory arousal index. Each point represents a patient. Dipper patients appear in green, and non-dipper patients appear in pink. The results are adjusted for confounding factors (age, sex, BMI, and antihypertensive medications). Abbreviations: AHI: apnea-hypopnea index; BMI: body mass index; BP: blood pressure; DF: estimated degrees of freedom; GAM: generalized additive model; OSA: obstructive sleep apnea; TSat90: time with oxygen saturation <90%.

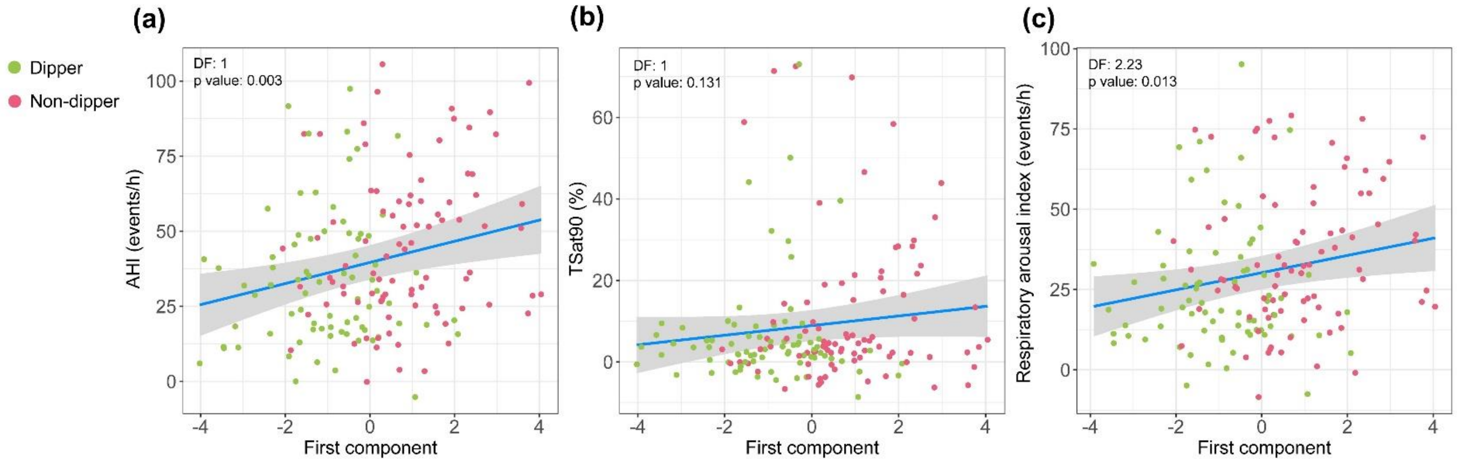

Figure S2. Metabolite set enrichment analysis including the metabolipidomic features identified as relevant to circadian BP control in OSA. Dot plot presenting the top 25 significantly enriched metabolite sets in which the identified metabolites and lipids may be involved. Each circle represents a metabolite set. The color gradient indicates the significance of the set according to p value, with yellow indicating higher p values and red indicating lower p values (y-axis). The size of the circle represents the enrichment ratio of the metabolite set, computed by observed hits / expected hits (x-axis).

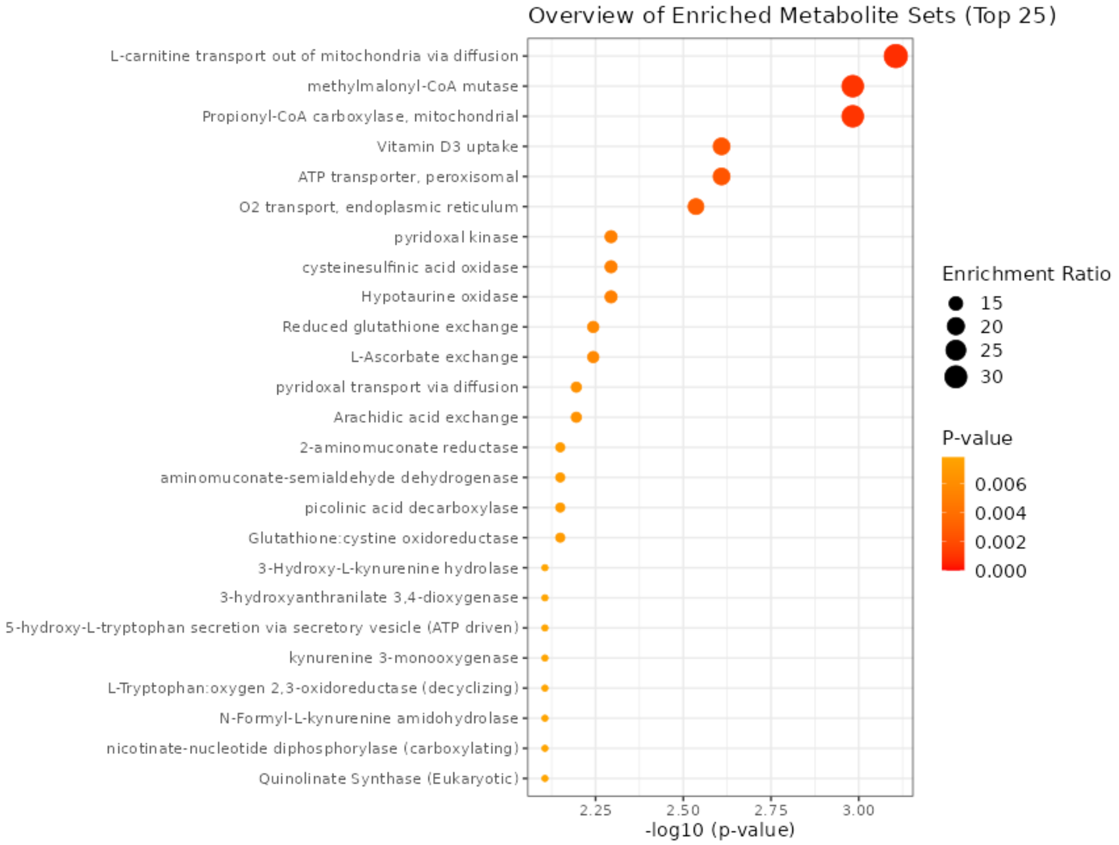

Table S1. Differentially expressed features between non-dipper and dipper patients in the metabolomic analysis. The results are adjusted for confounding factors (age, sex, BMI, and antihypertensive medications). Metabolites with FC <0.8 (downregulated) or >1.25 (upregulated) and p value <0.05 were considered. Features are represented as exact mass and RT. Definition of abbreviations: BMI = body mass index; FC = fold change; OSA = obstructive sleep apnea; RT = retention time.

Metabolomics		Ionization	FC	p-value
Molecular mass	RT (min)			
602.4646	11.71269	-	1.918	<0.001
827.1027	0.5837527	-	1.437	0.002
465.3101	8.988933	-	1.879	0.003
791.6017	11.42984	-	1.309	0.008
598.2597	8.905328	+	1.291	0.009
568.3252	9.260691	-	1.398	0.010
822.2788	11.38875	-	1.282	0.013
474.2918	12.98659	-	0.829	0.013
256.0955	4.755068	-	0.684	0.014
160.1219	0.5020314	-	0.738	0.015
370.1825	8.089659	-	0.774	0.017
760.4527	11.71748	-	0.788	0.018
240.1008	7.275254	-	0.693	0.020
816.2971	11.38298	-	1.628	0.022
449.3152	8.721428	-	1.527	0.022
499.2975	10.32784	-	1.456	0.025
318.26	11.57911	-	1.236	0.026
606.0906	12.08467	-	0.723	0.027
791.5651	10.62096	-	1.261	0.028
658.4229	11.58019	-	0.730	0.034
598.4338	11.2998	+	1.369	0.035
1108.853	11.57688	-	1.320	0.035
1025.551	13.29278	-	0.817	0.037
515.2295	11.71635	-	0.816	0.038
811.5371	14.02962	-	1.412	0.038
764.4844	12.07795	-	1.209	0.039
368.1674	8.3971	-	0.812	0.039
412.1364	11.15013	-	1.504	0.042
514.31245	10.82806	+	1.265	0.044
1212.914	11.70673	-	1.348	0.049
542.1265	8.394325	-	0.788	0.049

Table S2. Differentially expressed features between non-dipper and dipper patients in the lipidomic analysis. The results are adjusted for confounding factors (age, sex, BMI, and antihypertensive medications). Lipids with FC <0.8 (downregulated) or >1.25 (upregulated) and p value <0.05 were considered. Features are represented as exact mass and RT. Definition of abbreviations: BMI = body mass index; FC = fold change; OSA = obstructive sleep apnea; RT = retention time.

Lipidomics		Ionization	FC	p-value
Molecular mass	RT (min)			
166.9866	0.9055392	+	0.808	0.005
1249.356	9.835346	+	1.208	0.012
1338.199	10.34988	+	1.430	0.021
807.5784	7.231465	+	0.636	0.023
1171.264	7.82933	-	1.453	0.023
1079.255	7.688919	+	0.720	0.024
1266.198	10.65473	+	1.205	0.024
616.5038	8.262866	+	1.235	0.028
665.0966	5.214503	+	0.664	0.031
1428.382	10.15159	+	0.789	0.040
635.5499	7.994549	+	1.216	0.041
305.3186	2.950193	+	0.790	0.042
1384.454	9.951343	+	1.226	0.044

Table S3. Changes in the ABPM parameters after 6 months of OSA treatment with CPAP. Data are presented as the mean [95% CI]. P values <0.05 are presented in bold. Abbreviations: ABPM: ambulatory blood pressure monitoring; BP: blood pressure; CI: confidence interval; CPAP: continuous positive airway pressure; DR: dipping ratio.

	**OSA patients treated with CPAP**<br>**N = 84**
Dipping ratios	
24h DR	-0.02 [-0.04;0.01]
Systolic DR	-0.01 [-0.03;0.02]
Diastolic DR	0.02 [-0.01;0.04]
Nighttime BP	
Mean (mmHg)	**-3.49 [-6.30;-0.68]**
Systolic (mmHg)	-3.69 [-7.75;0.37]
Diastolic (mmHg)	-1.91 [-4.07;0.24]
Daytime BP	
Mean (mmHg)	-1.58 [-4.45;1.29]
Systolic (mmHg)	-2.99 [-6.45;0.46]
Diastolic (mmHg)	-1.15 [-3.35;1.04]
24h BP	
Mean (mmHg)	-2.12 [-4.49;0.25]
Systolic (mmHg)	**-4.00 [-7.33;-0.66]**
Diastolic (mmHg)	-1.44 [-3.19;0.31]
